# Differences in Functional Expression of Connexin43 and Na_V_1.5 by Pan- and Class-Selective Histone Deacetylase Inhibition in Heart

**DOI:** 10.3390/ijms19082288

**Published:** 2018-08-04

**Authors:** Xian Zhang, Dakshesh Patel, Qin Xu, Richard Veenstra

**Affiliations:** 1 Department of Pharmacology, State University of New York (SUNY) Upstate Medical University, Syracuse, NY 13210, USA; ZhangXi@upstate.edu (X.Z.); daksheshpatel10@gmail.com (D.P.); xuqinblue@gmail.com (Q.X.); 2 Department of Cell and Molecular Biology, SUNY Upstate Medical University, Syracuse, NY 13210, USA

**Keywords:** histone deacetylase inhibitors, class-selective, gap junction, cardiac sodium channel, connexin43, Na_V_1.5, cardiotoxicity

## Abstract

Class-selective histone deacetylase (HDAC) inhibitors were designed to improve safety profiles and therapeutic effectiveness in the treatment of multiple cancers relative to pan-HDAC inhibitors. However, the underlying mechanisms for their therapeutic and cardiotoxic potentials remain poorly understood. Cardiac sodium currents (I_Na_) and gap junction conductance (g_j_) were measured by whole cell patch clamp techniques on primary cultures of neonatal cardiomyocytes. Cardiac Na_V_1.5 sodium channel and connexin43 (Cx43) gap junction protein levels were assessed by Western blot analyses. Panobinostat produced concentration-dependent reductions in ventricular g_j_, peak I_Na_ density, and Na_V_1.5 protein expression levels. Membrane voltage (V_m_)-dependent activation of I_Na_ was shifted by +3 to 6 mV with no effect on inactivation. Entinostat (1 μM) did not affect ventricular g_j_, peak I_Na_ density, or I_Na_ activation. However, the I_Na_ half-inactivation voltage (V_½_) was shifted by −3.5 mV. Ricolinostat had only minor effects on ventricular g_j_ and I_Na_ properties, though I_Na_ activation was shifted by −4 mV. Cx43 and Na_V_1.5 protein expression levels were not altered by class-selective HDAC inhibitors. The lack of effects of class-selective HDAC inhibitors on ventricular g_j_ and I_Na_ may help explain the improved cardiac safety profile of entinostat and ricolinostat.

## 1. Introduction

Histone acetyltransferases (HATs) and histone deacetylases (HDACs) regulate the dynamic balance between acetylation and deacetylation of lysine residues of both histone and non-histone proteins. Non-histone proteins are involved in numerous critical cellular physiological functions, including apoptosis, autophagy and DNA damage repair [1]. HDACs have been suggested to be overexpressed in tumor cells and alter the expression and function of many tumor suppressor genes like p53 [2,3]. However, the four U. S. Food and Drug Administration (FDA) approved pan-HDAC inhibitors are limited to non-solid tumors and have been reported to cause serious cardiotoxicities such as QT interval prolongation, ventricular arrhythmia and unexpected sudden cardiac death [4,5,6,7]. In order to improve their effectiveness on solid tumors as well as to minimize their cardiac side effects, a new generation of HDAC inhibitors, class-selective HDAC inhibitors were designed and developed [8]. Significantly, some of them have shown promising therapeutic effects against leukemias as well as breast and lung cancers with less cardiotoxicities [9,10,11,12]. However, the mechanisms behind their therapeutic and lack of cardiotoxic potentials is still unclear.

Our previous studies have suggested the involvement of the inward depolarizing cardiac sodium current (I_Na_) and connexin43 (Cx43) on pan-HDAC inhibitor induced cardiac side effects [13,14]. Physiologically, Cx43, the predominant gap junction protein in the ventricles, is required for synchronous longitudinal and transverse conduction of cardiac action potentials throughout the heart [15]. Furthermore, cardiac I_Na_ through the voltage-gated cardiac sodium channel (Na_v_1.5) is vital for normal cardiac electrical activity [16]. Gain or loss of function of this protein is related to long QT syndrome or Brugada syndrome [17]. Previous research in our laboratory has indicated that both the protein expression of Cx43 and Na_v_1.5 and their resultant electrophysiological currents were reduced by pan-HDAC inhibition [13,14]. These functional reductions in rapid depolarization and electrical coupling likely contribute to decreased excitability and slowed conduction, thereby increasing the vulnerability to reentrant arrhythmias. Since entinostat (MS-275), a class I selective HDAC inhibitor, apparently causes minimal cardiotoxic effects [7,8,18], we hypothesize that class-selective HDAC inhibition does not cause the functional downregulation of the cardiac I_Na_ or gap junction conductance (g_j_) observed with the first two FDA-approved pan-HDAC inhibitors, vorinostat (VOR, Zolinza^®^) and romidepsin (FK228, Istodax^®^) [13,14]. In this study, we examine the effects of another FDA-approved HDAC inhibitor, panobinostat (LBH589, Farydak^®^), and two clinical trial class-selective HDAC inhibitors, entinostat (MS-275, HDAC class I selective), and ricolinostat (ACY-1215, HDAC class IIb selective), on Cx43-mediated cardiac gap junction coupling and excitatory cardiac sodium current density mediated primarily by Na_V_1.5.

## 2. Results

### 2.1. Inhibition of Cardiac HDAC Activity by Pan- and Class-Selective HDAC Inhibitors

**Figure 1:**
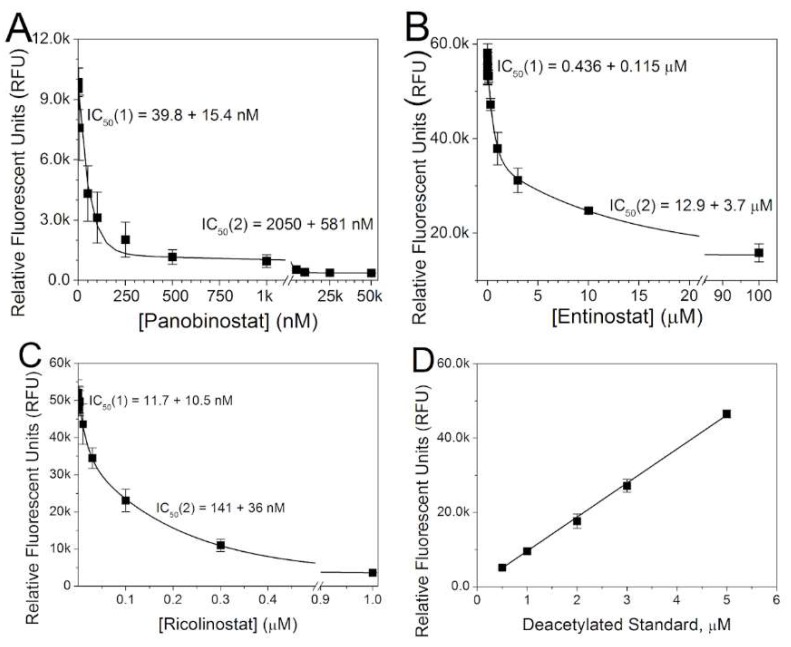
Histone deacetylase (HDAC) activity assays for pan- and class-selective HDAC inhibitors. (**A**) The total HDAC inhibition curve for neonatal mouse ventricular cardiomyocytes treated for 18–24 h with increasing concentrations of panobinostat. Two IC_50_s of approximately 40 and 2000 nM are apparent. (**B**) The cardiomyocyte HDAC inhibition curve for the class I HDAC selective inhibitor entinostat. Again, the HDAC activity curve was best fit with a double exponential function. (**C**) The ventricular cardiomyocyte HDAC inhibition curve for the HDAC6 class IIb HDAC inhibitor ricolinostat exhibits two distinct IC_50_s for total HDAC activity. (**D**) The standard curve for the deacetylated fluorescent Fluor-de-Lys product showing a linear response. All Fluor-de-Lys HDAC activity assays were performed in triplicate and the average ± SEM values are shown. The parameters for the double exponential fits of the dose-response curves are listed in Table 1.

The inhibitory action of panobinostat, entinostat, and ricolinostat on total HDAC activity in cultured ventricular cardiomyocytes was assessed using the fluorometric Fluor de Lys assay as performed in our previous studies [13,14]. As with Trichostatin A (TSA), vorinostat (VOR), and romidepsin (FK228), the inhibitory dose-response curves for the three HDAC inhibitors used in this study were best described by a double exponential decay curve, indicative of two inhibitory sites with a >10-fold difference in the apparent IC_50_s between the high and low affinity HDAC inhibitory sites (Figure 1). The fluorometric data was fitted with the equation RFU = A_1_·exp(−[HDACI]/C_1_) + A_2_·exp(−[HDACI]/C_2_) + B where A_X_ = the amplitude of each exponential component, [HDACI] = HDAC inhibitor concentration, C_X_ = the decay constant for each exponential component, B = the background fluorescence count constant, RFU = relative fluorescence units = total counts for each sample, and the IC_50_ = 0.693·C_X_ for each apparent inhibitory site. The fit parameters for all HDACIs examined thus far are listed in Table 1.

Thus far, all HDAC inhibitors tested on cultured neonatal mouse ventricular cardiomyocytes (NMVMs) exhibit two apparent IC_50_s for total HDAC activity, which we hypothesize corresponds to the different classes (I, II, IV) and subclasses (IIa/b) of zinc-dependent HDACs. Entinostat is reported to inhibit HDACs 1 and 3 with an IC_50_ <1 μM and exhibits incomplete inhibition of HeLa nuclear extract HDAC activity with an IC_50_ of 11 μM [19]. Our apparent high and low affinity IC_50_s for total cardiac HDAC activity of 0.43 and 12.9 nM are in close agreement with these IC_50_ values. Ricolinostat possesses >10-fold selectivity for HDAC6 over HDACs 1, 2, and 3 with IC_50_s of approximately 5 and 50 nM, respectively [20]. Our observed IC_50_s of 12 and 140 nM are approximately 2 times higher than those reported IC_50_s, but are otherwise consistent with HDAC6 and HDAC1-3 inhibition.

### 2.2. Effects of Class-Selective HDAC Inhibitors on Gap Junction Coupling

**Figure 2:**
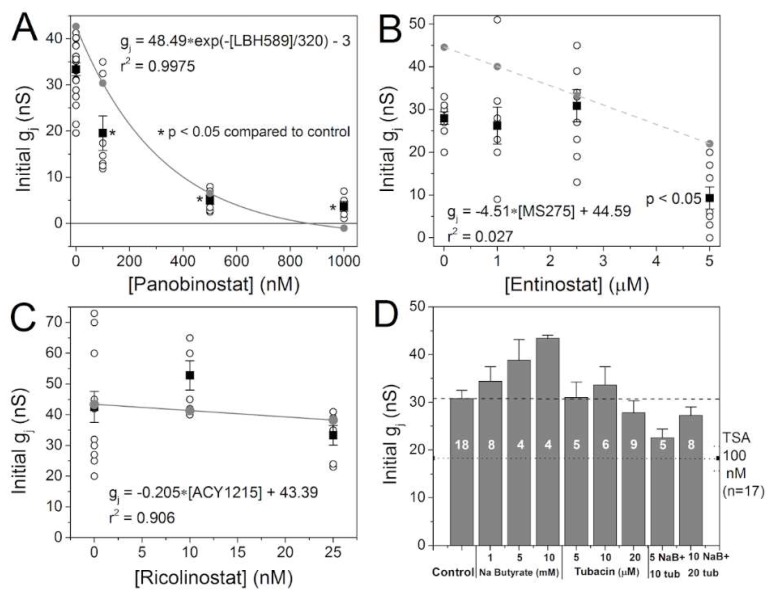
Effects of pan- and class-selective HDAC inhibition on gap junction coupling. (**A**) The gap junction conductance (g_j_) was measured between neonatal mouse ventricular cardiomyocytes (NMVMs) treated with 0, 100, 500, or 1000 nM panobinostat for 18–24 h in culture. Pan-HDAC inhibition reduced g_j_ in a concentration-dependent manner. (**B**) Class I HDAC selective inhibition with entinostat had no effect on ventricular g_j_ except at the highest, and least selective, concentration tested. (**C**) Ricolinostat, a class IIb HDAC6-selective inhibitor produced a slight, but non-significant, increase and then decrease in ventricular g_j_ as the concentration was increased from 10 to 25 nM. (**D**) The effect of a weak HDAC class I/IIb inhibitor, sodium butyrate (NaB), and the prototype HDAC6 inhibitor, tubacin, on g_j_ were tested independently and in combination [21,22,23]. NaB non-significantly increased g_j_ in a concentration-dependent manner while tubacin (tub) produced a non-significant increase and then decrease in g_j_ with increasing concentration. When concentrations of NaB and tubacin that increased g_j_ by 26% and 9% respectively were applied in combination, g_j_ decreased by 26%, indicating an opposite and negative response when class I and IIb HDAC inhibition is combined.

Previous results with three pan-HDACIs consistently showed a dose-dependent decrease in cardiac ventricular gap junction conductance (g_j_) [14]. To assess whether this observation holds true for panobinostat and if class I and IIb HDAC inhibition has similar effects, g_j_ was measured in NMVM cell pairs after 18–24 h treatment with varying concentrations of panobinostat, entinostat, and ricolinostat. Consistent with previous findings, pan-HDAC inhibition by panobinostat produced a significant dose-dependent decrease in ventricular g_j_ (Figure 2A). The concentration-dependent decline in g_j_ by panobinostat was best fit by an exponentially decaying function with an amplitude of 48.5 and exponential decay constant of 320 nM (gray points and curve). By contrast, class I HDAC-selective inhibition by 1 or 2.5 μM entinostat did not reduce g_j_, though a significant reduction in g_j_ was obtained with 5 μM entinostat, 10-fold higher than the observed high affinity IC_50_ that likely corresponds to the class I-selective HDAC inhibition (Figure 2B). Linear regression fits correlated poorly with the g_j_ – [entinostat] relationship (gray points and dashed gray line). Class IIb HDAC inhibition with 10 or 25 nM ricolinostat produced modest but non-significant increases and decreases in g_j_, respectively (Figure 2C). Linear regression analysis of this data produced a straight line with a slope of only −0.2 ns/nM ricolinostat (gray points and line). These results suggest that class-selective HDAC inhibition does not affect ventricular g_j_ as dramatically as pan-HDAC inhibition, and even then only produces downregulatory effects only when HDACI concentrations were high enough to begin to exceed the HDAC class-selective properties of entinostat and ricolinostat.

Prior to the development of entinostat and ricolinostat, we explored the combinatorial effect of class I and IIb HDAC inhibition using sodium butyrate and the prototypical HDAC6 inhibitor, tubacin [21]. Sodium butyrate (NaB) is a short chain fatty acid non-specific HDAC inhibitor with mM affinities for the class II HDACs, even weaker and incomplete inhibitory activity of class IIa HDAC isoforms, and no activity against the class IIb HDAC6 [22,23]. 4-phenylbutyrate (4-PB) belongs to this group of HDAC inhibitors and is known to upregulate Cx43 expression in human glioblastoma and embryonic kidney cells [24,25]. The HDAC inhibitory profiles of the HDAC inhibitors used in this study are listed in Table 2. The HDAC inhibition assays from the cited studies were performed using different reagents, but the highlighted columns indicate HDAC activities measured using the Fluor-De-Lys^®^ substrate on recombinant human HDACs [23]. Sodium butyrate increased ventricular g_j_ in a dose-dependent manner while tubacin exhibited a biphasic response, causing a slight increase in g_j_ or no effect at low doses, followed by a slight decrease at the highest tested dose of 20 μM (Figure 2D). However, when we combined 5 mM NaB with 10 μM tubacin, concentrations of these two HDAC inhibitors that increased g_j_ slightly when applied individually, the opposite effect occurred. The reduction in g_j_ when combining 5 mM NaB and 10 μM tubacin was 25% relative to control g_j_ values and 40% relative to 5 mM NaB, suggesting that combinatorial class I and IIb HDAC inhibition negatively affects ventricular gap junction coupling while inhibition of either of these two classes of HDACs alone has negligible effects.

### 2.3. Effects of Class-Selective HDAC Inhibitors on Peak Cardiac Na^+^ Current Density

**Figure 3:**
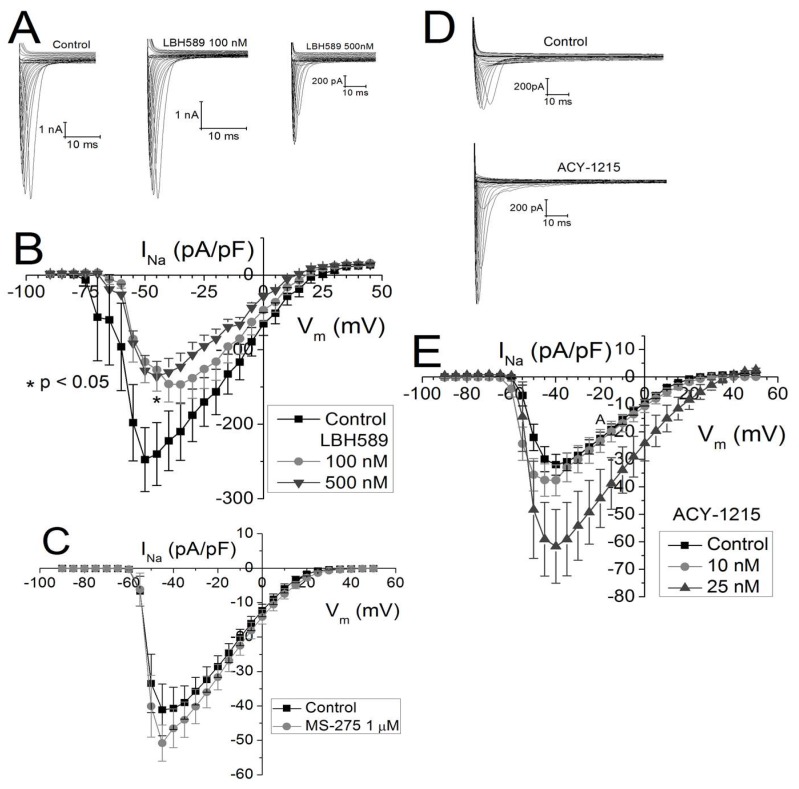
Peak cardiac I_Na_ effects of pan- and class-selective HDAC inhibition. (**A**) Families of ventricular cardiomyocyte I_Na_ current traces produced in response to depolarizing voltage clamp steps from a holding potential of −120 mV before and after treatment with 100 or 500 nM panobinostat (LBH589). (**B**) Average I_Na_ current–voltage (I–V) relationships from control (*n* = 8) and LBH589 treated cells (*n* = 8, 10) illustrating a concentration-dependent decrease in peak I_Na_ density (pA/pF) with pan-HDAC inhibition. (**C**) Average I_Na_–V_m_ relationships for control (*n* = 9) and 1 μM entinostat (MS-275, *n* = 8) treated NMVMs indicates that class I HDAC inhibition does not affect the cardiac Na^+^ current density. (**D**) Representative I_Na_ current traces from control and ricolinostat (ACY-1215) treated NMVMs showing an increase in peak I_Na_ with 25 nM ACY-1215. (**E**) Average I_Na_–V_m_ relationships for control (*n* = 17) and 10 and 25 nM ricolinostat (*n* = 8, 7) exhibit a concentration-dependent, but non-significant, increase in peak I_Na_ density with class IIb HDAC6 inhibition.

As with gap junction coupling, previous results with pan-HDAC inhibitors have revealed a significant reduction in sodium current density [13]. Thus, we examined the effect of panobinostat on the cardiac Na^+^ current density in NMVMs. True to form, panobinostat decreased the cardiac sodium current density in a dose-dependent manner (Figure 3A,B). Contrary to pan-HDAC inhibition, class I HDAC inhibition with 1 μM entinostat had no significant effect on the peak Na^+^ current density (Figure 3C). Surprisingly, the opposite effect was seen with HDAC6 inhibition by 25 nM ricolinostat (Figure 3D,E). Despite the apparent 93% increase in peak I_Na_ with 25 nM ricolinostat, the mean values did not achieve statistical significance (*p* = 0.054, one-way ANOVA).

### 2.4. V_m_-Dependence of g_Na_ Activation and Inactivation

**Figure 4:**
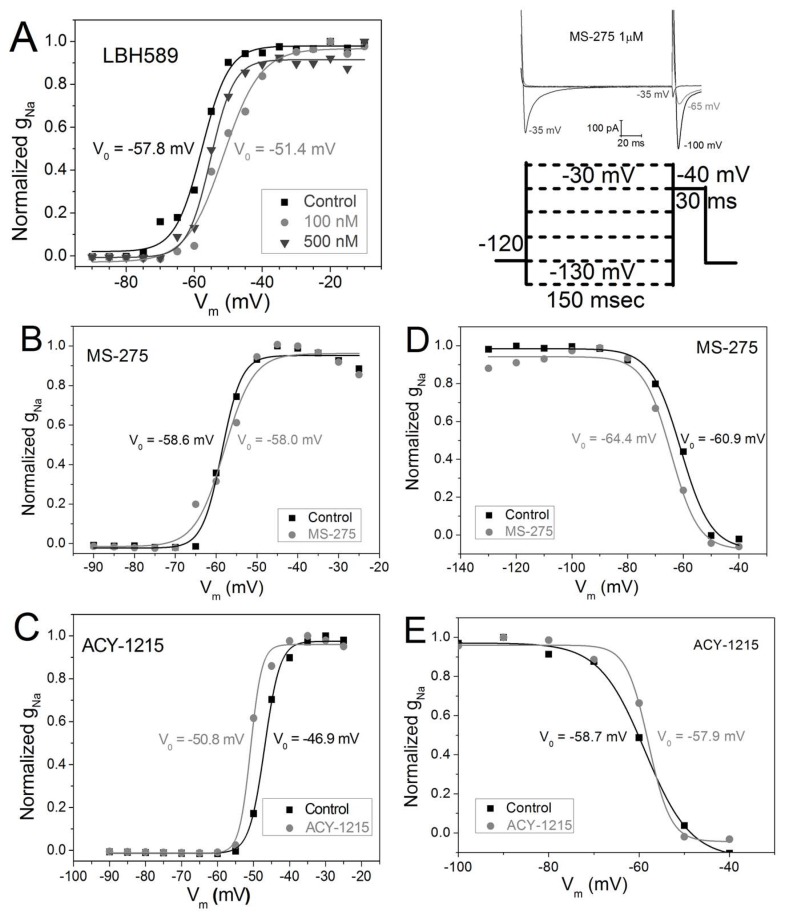
Effect of HDAC inhibition on V_m_-dependent I_Na_ activation and inactivation. (**A**) V_m_-dependent steady state g_Na_ activation curves for the experiments shown in Figure 3B illustrating a slight positive shift in the half activation voltage (V_½_) of +2.5–6.4 mV with pan-HDAC inhibition by panobinostat. (**B**) There was no shift in the g_Na_–V_m_ activation curve for class I selective HDAC inhibition by 1 μM entinostat. (**C**) HDAC6 inhibition by 25 nM ACY-1215 produced a −4 mV shift in the g_Na_–V_m_ activation curve. (**D**) V_m_-dependent inactivation was examined using a prepulse protocol and 1 μM entinostat caused a −4 mV shift in the g_Na_ steady state inactivation (*n* = 9 (control), 8). (**E**) Ricolinostat (25 nM, *n* = 7) did not produce a shift in the g_Na_ V_½_ relative to control values (*n* = 6).

Since both the activation and inactivation of I_Na_ are dependent on the membrane potential (V_m_), we calculated the normalized Na^+^ conductance (g_Na_) by dividing the average peak I_Na_ by the difference between V_m_ and the reversal potential (E_rev_) for I_Na_, and then divided the g_Na_ value for each V_m_ by the maximum g_Na_ value for each experiment. The g_Na_–V_m_ curves were fit with a Boltzmann function gNa= gNamax+ (gNa− gNamax)[1+exp((Vm− V1/2)dVm)] using Origin 8.6. A positive shift in the half activation voltage (V_½_) of +2 to +8 mV was seen with trichostatin A, vorinostat, and romidepsin in previous experiments [13]. A maximum positive shift in the g_Na_ activation curve of +6.4 mV was observed with 100 nM panobinostat (Figure 4A), again consistent with previous findings using pan-HDAC inhibitors. No shift in the activation threshold is apparent in the entinostat I–V relationship and the g_Na_ activation curve substantiates this observation (Figure 3C and Figure 4B). In contrast to the other HDAC inhibitors, 25 nM ricolinostat shifted the I_Na_ activation threshold and the g_Na_ V_½_ by approximately −4 mV (Figure 3E and Figure 4C). Previously, no shift in the V_m_-dependent g_Na_ inactivation curved was observed trichostatin A, vorinostat, or romidepsin [13]. The g_Na_ inactivation protocol was not performed on panobinostat treated NMVMs, but the V_½_ for inactivation was shifted by −3.5 mV in the presence of 1 μM entinostat. No shift in the g_Na_ inactivation curve was observed with 25 nM ricolinostat. To summarize, pan-HDAC inhibition by panobinostat shifted the V_½_ for activation, consistent with previous pan-HDACIs. Class I HDAC inhibition by 1 μM entinostat had no effect on activation but shifted inactivation in the hyperpolarizing direction by 3–4 mV and HDAC6 inhibition with ricolinostat shifted activation approximately 4 mV negative while having no effect on inactivation.

### 2.5. Cardiac Late I_Na_

**Figure 5:**
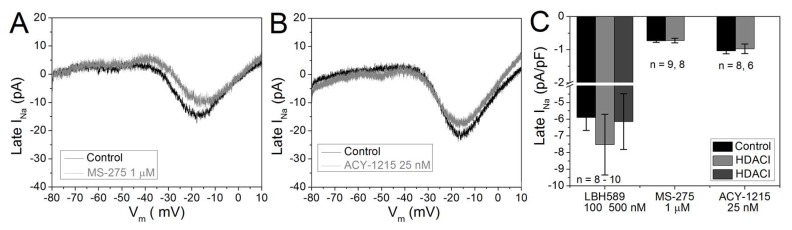
HDAC inhibition does not increase late I_Na_. (**A**) Whole cell late I_Na_ currents generated by a 90 mV, 3.6 s V_m_ ramp from −80 to +10 mV in a control NMVM and a NMVM treated with 1 μM entinostat for 18–24 h. (**B**) Late I_Na_ currents recorded from a control NMVM and a NMVM treated with 25 nM ricolinostat overnight. (**C**) Averaged late I_Na_ current density (pA)/pF) from 6–10 NMVMs treated with panobinostat, entinostat, or ricolinostat illustrating no increase in late I_Na_ relative to untreated NMVMs after 18–24 h exposure to pan- or class-selective HDAC inhibitors. Late I_Na_ was measured as the average steady state I_Na_ current from 100 to 150 msec in control and panobinostat treated NMVMs in response to activating V_m_ pulses (Figure 3A,B).

Increases in late or persistent I_Na_ have been linked to cardiac arrhythmias and heart failure [27]. To examine the effects of HDAC inhibitors on late I_Na_, we either measured the average I_Na_ from 100 to 150 ms in response to the I_Na_ activating V_m_ steps or applied a slow (40 msec/mV) V_m_ ramp from −80 to +10 mV from a holding potential of −60 mV. Using the averaged inward whole cell current from 100 to 150 msec, no difference in late I_Na_ was observed after overnight treatment with 100 or 500 nM panobinostat compared to control I_Na_ recordings, again consistent with our previous pan-HDAC inhibitor results (Figure 5C). Subsequently, we modified a voltage clamp ramp protocol designed to generate late I_Na_ I–V relationships [28]. Late I_Na_ currents recorded from control, 1 μM entinostat, and 25 nM ricolinostat treated NMVMs are shown in Figure 5A,B. Again, no increase in late Na^+^ current density was observed with class I or IIb HDAC inhibition (Figure 5C).

### 2.6. Cx43 and Na_V_1.5 Expression

**Figure 6:**
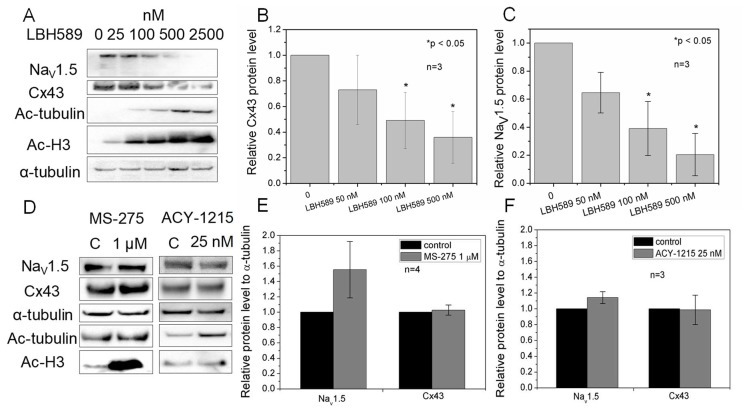
Changes in cardiac Cx43 and Na_V_1.5 protein levels by panobinostat, entinostat and ricolinostat. (**A**) Representative Western blot of lysed NMVMs treated with 0, 25, 100, 500, and 2500 nM panobinostat for 24 h. Ac-tubulin is acetylated α-tubulin, Ac-H3 is acetylated histone 3, and α-tubulin was used as a loading control. (**B**) Densitometry scans of Cx43 Western blots (*n* = 3) to quantify the reduction in Cx43 protein levels with increasing concentrations of panobinostat (* *p* < 0.05). (**C**) Densitometry scans of Na_V_1.5 Western blots (*n* = 3) quantifying the statistically significant decrease in Na_V_1.5 protein levels with higher concentrations of panobinostat (* *p* < 0.05). (**D**) A representative Western blot of lysed ventricular myocytes treated with 1 μM entinostat (MS-275) or 25 nM ricolinostat for 24 h. Note that MS-275 only increased the Ac-H3 signal while ACY-1215 only increased the Ac-α-tubulin signal, consistent with their class I and HDAC6 inhibitory activities. (**E**) Densitometry scans of 1 μM entinostat Western blots (*n* = 4) illustrating no significant changes in Cx43 or Na_V_1.5 protein levels with entinostat, a class I HDAC-selective inhibitor. (**F**) Densitometry scans of 25 nM ricolinostat Western blots (*n* = 3) illustrating no significant changes in Cx43 or Na_V_1.5 protein levels with ricolinostat.

Because panobinostat caused a concentration-dependent reduction in I_Na_ and g_j_, we performed Western blot analyses on NMVMs to determine if there were any alterations in cardiac Na_V_1.5 and Cx43 protein expression level induced by pan-HDAC inhibition. Protein expression analysis of cultured NMVMs revealed a concentration-dependent decrease of both Na_V_1.5 and Cx43 with panobinostat treatment (*p* < 0.05, *n* =3), whereas no changes were observed with entinostat and ricolinostat (Figure 6). Acetyl-α-tubulin and acetyl-histone 3 were used as cytoplasmic and nuclear acetylation markers respectively. α-tubulin was used as a loading control.

## 3. Discussion

The HDAC inhibitors have tremendous potential in the field of cancer pharmacology due to their involvement in key regulatory and gene expression pathways [29,30]. To date, four non-selective HDAC inhibitors, vorinostat (VOR, suberoylanilide hydroxamic acid (SAHA), Zolinza™, 2006), romidepsin (FK228, depsipeptide, Istodax™, 2009), belinostat (PXD101, Beleodaq™, 2014) and panobinostat (LBH589, Farydak™, 2015), have been approved by FDA for chemotherapy of cutaneous/peripheral T-cell lymphoma or multiple myeloma. Furthermore, hundreds of HDAC inhibitors are undergoing clinical trials (www.clinicaltrials.gov). However, HDAC inhibitor-related severe cardiotoxic side effects, especially QT interval prolongation, ventricular arrhythmia and unexpected sudden cardiac death, necessitated clinical trial termination or dose readjustments of numerous promising non-selective HDAC inhibitors [4,5,6,7]. In addition, panobinostat, the most potent pan-HDAC inhibitor, carries a black box warning for severe cardiac abnormalities on its prescription label [31]. Notably, currently approved HDAC inhibitors can non-selectively inhibit all 11 zinc-dependent isoforms in this family [32]. Based on their sequence homology to *Saccharomyces cerevisiae* HDACs and their cellular location, those targeted HDACs can be divided into four classes. Class I HDACs (HDAC1, 2, 3 and 8) are homologous proteins of yeast that reduce potassium-dependent 3 (Rpd3). These HDACs are mainly present in the nucleus and are ubiquitously expressed. Class II HDACs share sequence homology to yeast histone deacetylase 1 (Hda1). They are further divided into two groups based on their subcellular localization, with class IIa (HDAC4, 5, 7 and 9) shuttle between the nucleus and cytoplasm, while class IIb (HDAC6 and 10) is located in the cytoplasm. HDAC11 is the only class IV HDAC that shares sequence similarity with Rpd3 and Hda1 and has not been well studied.

HDACs are tissue specific. For example, class I HDACs are ubiquitous, while class II HDACs are only expressed in specific tissues such as heart, kidney, and brain. Furthermore, different HDACs are overexpressed in different cancers. It is worth noting that the different classes of HDAC have distinct regulatory mechanisms and play distinct roles on cardiac activities. For example, class IIa HDACs are regulated by the calcium/calmodulin-dependent protein kinase II (CaMKII) pathway and inhibits MEF (myoblast-enhancing factor)-associated cardiac hypertrophy and is therefore used as a protective agent for cardiac hypertrophy [33]. Class I HDACs play a “pro-hypertrophic” role in heart and inhibition of this HDAC class attenuates cardiac hypertrophy through tuberous sclerosis complex 2-dependent mTOR repression [34]. Based on these facts, it has been hypothesized that class-selective HDACs present a new strategy for the treatment of cancer, with the goal of minimizing cardiotoxicity [8,35]. Encouragingly, the development of the Class I HDAC inhibitor entinostat has enabled this goal. Entinostat combined with exemestane increased overall survival and progression-free survival in patients with advanced hormone receptor (HR)-positive breast cancer without adverse cardiac toxicity events [7,11]. But the mechanisms for the reduced arrhythmogenic cardiotoxicities observed with pan-HDAC inhibitors remains essentially unknown.

Blockade of human ether-a-go-go (hERG) channels and the subsequent inhibition of the rapidly activating delayed rectifier potassium current (I_Kr_) is responsible for more than 95% of acquired or drug-induced Long QT syndromes and led to withdrawal of multiple drugs including terfenadine (Seldane) [36]. Therefore, hERG-dependent assays were required for screening new therapeutic drugs according to the preclinical testing recommendations of the ICH S7A guidelines. All four FDA approved HDAC inhibitors, with the possible exception of panobinostat, failed to show significant hERG blockade activity, implying that other mechanisms are involved in HDAC inhibitor-related cardiotoxicities [18,37,38]. In addition, the three HDAC inhibitors in this study showed insignificant effects on steady state and transient outward K^+^ currents of NMVM cells (Supplementary Figures S1 and S2). Thus, we hypothesize that hERG is not responsible for cardiac side effects of HDAC inhibitors and, hence, we focused our studies on other major cardiac ionic currents which can be altered by HDAC inhibition.

After examining four pan-HDAC inhibitors including panobinostat, we have consistently observed concomitant reductions in gap junction coupling and peak I_Na_, without alterations in I_Na,Late_ and a slight shift of 2–3 mV during activation with no change in inactivation of I_Na_ [13,14]. With the phase 3 clinical trial class I HDAC inhibitor, entinostat, we observed no change in ventricular g_j_, peak I_Na_ density, or the V_½_ for V_m_-dependent activation and inactivation at the class-selective concentration of 1 μM. The lack of these effects may help explain the safer record of entinostat pertaining to cardiotoxicity, especially considering that this concentration is still more than three times the therapeutic dose used in clinical trials [7,18]. The phase 1–2 clinical trial class IIb HDAC inhibitor, ricolinostat, also had minor effects on ventricular g_j_ and I_Na_ electrophysiological properties. A 1 × IC_50_ dose of ricolinostat produced a modest increase in g_j_ while a 2.5 × IC_50_ dose produced a similar decrease in g_j_. This trend (induction at lower concentration and reduction at higher concentration) was also observed with the prototype HDAC6 inhibitor, tubacin, although none of the g_j_ changes were significant. It is worth noting that 1 × IC_50_ dose of ricolinostat is still four times higher than the maximum dose used in clinical trials. Thus, we conclude that therapeutic doses of ricolinostat are unlikely to cause any significant changes in gap junction coupling. In addition, the differential effects of pan-, class 1-selective, and class IIb-selective HDAC inhibitors implies that different classes of HDACs likely have different effects on Cx43 expression and function. Furthermore, the combined effects of sodium butyrate and tubacin suggest that inhibiting multiple classes of HDACs accounts for some of the adverse cardiac side effects attributable to pan-HDAC inhibitors.

HDAC6 is preferentially inhibited by ricolinostat at concentrations applied. One of its deacetylated substrates is Hsp90, the 90 kDa heat shock protein [39,40]. Hsp90 is an ATP-dependent chaperone that helps stabilize many proteins. Hsp90 has been shown to serve two distinct roles relevant to Cx43 expression and function. Hsp90 mediates the mitochondrial translocation of Cx43 and increases the mitochondrial membrane expression of Cx43 at the expense of cell surface expression of Cx43 [41]. Hsp90 is also part of a Cx43 promotor P2 region protein complex and is involved in Ras-mediated Cx43 upregulation [42]. Thus, partial or complete inhibition of HDAC6 deacetylase activity by ricolinostat will result in increased acetylation of Hsp90 and promote the disparate actions of Hsp90 on Cx43 expression and localization. In addition, microtubules are responsible for Cx43 forward trafficking and consist of α-tubulin and β-tubulin [43]. α-tubulin is another known substrate for HDAC6. siRNA mediated knockdown of HDAC6 or non-selective HDAC inhibition induces tubulin hyper-acetylation, which was accompanied by aggregate formation in cardiomyopathy mice hearts [44]. However, a recent structural analysis revealed that the lysine residue K40 of α-tubulin targeted by HDAC6 deacetylation resides on the inner face of microtubules, and that K40 acetylation status had no effect on the ultrastructure of microtubules [45].

Entinostat preferentially inhibits HDAC1 by two-fold over HDAC3 and 10–100-fold over other HDACs. Two laboratories, including ours, have demonstrated that HDAC1 is bound to the Cx43 promoter region [14,46]. Loss of HDAC1 drastically increases gene silencing, including *GJA1*, resulting in decreased Cx43 mRNA levels [47]. Therefore, HDAC inhibitors capable of HDAC1 inhibition, e.g., pan-HDAC and class I-selective HDAC inhibitors, should cause a reduction of Cx43 expression. We did not observe any change in Cx43 protein level with 1 μM entinostat, which may result from the compensatory deacetylase effects of class IIa HDACs. HDAC4 and HDAC5 colocalize with Cx43 and a class IIa HDAC inhibitor MC1568 produced Cx43 hyperacetylation and dissociation from intercalated disk gap junctions in ventricular cardiomyocytes [48]. This may also explain the observation that non-selective inhibition of class I and II HDACs by pan-HDAC inhibitors significantly reduces Cx43 expression, but not by a class I HDAC inhibitor.

Finally, we observed a moderate, but not significant, increase in peak I_Na_ at twice the HDAC6 IC_50_ concentration of ricolinostat. The cardiac sodium channel Na_V_1.5 protein is acetylated and acetylation of K on the III-IV linker reduces its forward trafficking to the cell surface, thus reducing cardiac Na^+^ current density [13,49]. The HDACs associated with Na_V_1.5 are not yet identified, but our results suggest that HDAC6 activity may influence the surface expression and stability of the Na_V_1.5 protein complex either by directly modulating the acetylation of Na_V_1.5 or indirectly by modulating the acetylation, phosphorylation, or ubiquitination of Na_V_1.5 interacting proteins. Mutations and expression of key Na_V_1.5 interacting proteins like α1-syntrophin, calveolin-3, plakophilin-2, and others are known to influence the expression and function of the Na_V_1.5 channel and are linked to increased risk for cardiac arrhythmias and sudden cardiac death [16]. Overall, the class-selective HDAC inhibitors exhibit no or moderate effects on Cx43-mediated gap junction communication and cardiac sodium current density, unlike pan-HDAC inhibitors, which may help explain their lack of cardiotoxicity and improved safety profile.

## 4. Material and Methods

### 4.1. Cell Culture

Neonatal mice born from an inbred C57BL/6 mouse colony were anesthetized using isoflurane and the hearts were excised in accordance with procedures approved by the SUNY Upstate Medical University Institutional Animal Care and Use Committee (IACUC) # 263 on 15 March 2017. The excised hearts were separated into atria and ventricles and exposed to collagenase solution for digestion. The dissociated cells were pre-plated for 30 min to reduce fibroblast content by differential adhesion and the supernatant was collected, centrifuged and re-suspended in 1 mL/heart of M199/10% FBS culture media. The primary cells were plated into 96-well plates for HDAC activity assays, 35 mm culture dishes for patch clamp electrophysiology, or 60 mm culture dishes and homogenized after 3 days for Western blots.

### 4.2. HDAC Inhibitors

Panobinostat (LBH589), entinostat (MS275) and ricolinostat (ACY-1215) were purchased from Selleck Chemicals (Houston, TX, USA), dissolved in DMSO, and stored at −20 °C. The 100-mM DMSO solutions of LBH589, MS-275 and ACY-1215 were diluted to the desired experimental test concentrations in M199 and applied to NMVM overnight for 18–24 h before experimental procedures. Final DMSO levels were <0.005% (vol/vol).

### 4.3. HDAC Activity Assay

Aliquots of 2 × 10^5^ ventricular myocytes per well (96-well plate) were grown in 200 μL of 200 μM bromodeoxyuridine (BrDU)/M199, exchanged daily. Cell densities were counted with a hemocytometer and seeded into the wells. Wells were incubated with 2000 pmoles of the acetylated Fluor-de-Lys^®^ substrate for 8 h during HDAC inhibition [13,14]. Cells were incubated with varying doses of HDAC inhibitor, panobinostat, for 24 h. Protocols for HDAC activity assay were developed according to manufacturer’s directions (Cat. # BML-AK500, Enzo Life Sciences, Farmingdale, NY, USA) and background subtracted relative fluorescence unit (RFU) counts were acquired with a BIO-TEK Synergy 2 plate reader (360 nm excitation, 460 nm emission).

### 4.4. Dual Whole Cell Patch Clamping

Gap junction currents (I_j_) were recorded in the dual whole cell configuration according to previously published methods [50]. Upon establishing a dual whole cell patch, I_j_ was recorded using a 30 s, 20 mV trans-junctional voltage protocol. All dual whole cell current recordings were low-pass filtered at 500 Hz and digitized at 2 KHz using pClamp 8.2 and graphical analysis performed using Origin 7.5 or 8.6 software as previously described [14].

### 4.5. Whole Cell Patch Clamping

Single whole cell patch electrode voltage clamp experiments were performed on neonatal mouse ventricular myocytes (NMVMs) using conventional procedures with an Axon Instruments Axopatch 1D or 200B patch clamp amplifier, Digidata 1320A or 1440 A/D converter, and pClamp8.2 or 10.1 software (Molecular Devices, San Jose, CA, USA). Transient (peak) and steady state outward potassium (I_K,to_ and I_K,ss_) currents were recorded during 1 sec voltage steps from a holding potential (V_h_) of −100 to +60 mV in 10 mV increments. Voltage-gated sodium currents (I_Na_) were elicited from a V_h_ of −120 mV during voltage steps from −90 to +50 mV in 5 mV increments for 150 ms using reduced NaCl solutions. For the I_Na_ inactivation protocol, V was −120 mV and the prepulse voltage increased from −130 to −30 mV in +5-mV increments for 150 ms followed by a 30-ms activation step to −40 mV [13]. Late I_Na_ protocol was measured as the average current from 100 to 150 ms of the activating voltage steps or were recorded during a ramp from −80 to +10 mV in 0.1 mV/4 ms steps from a V_h_ of −60 mV [13,28].

### 4.6. Western Blot

Ventricular myocytes were cultured at high density in 35 mm culture dishes for four days in 3 mL of BrDU/M199 media, harvested, and lysed with 1% Triton X-100 extraction buffer (50 mM Tris pH 8.0, 150 mM NaCl, 0.02% Sodium azide, 1.0 mM PMSF, 1 μg/mL Aprotinin, 1% Triton X-100, 1 mM Na_3_VO_4_, 50 mM NaF) with protease inhibitors (Roche Life Sciences, Branford, CT, USA). One dish from each primary culture served as a control sample and a second dish was treated with panobinostat or entinostat for 24 h prior to harvesting. Sonicated samples (three 30 sec pulses) were incubated on ice for 30 min, centrifuged at 14,000 rpm (10 min at 4 °C), transferred to new tubes, and protein concentrations were measured using the coomassie blue protein assay (Bio-Rad, Hercules, CA, USA). Total protein/sample was heated (55 °C) and loaded onto an SDS-PAGE gel and electrophoresed for 90 min at 110 V in 4× Nupage sampling buffer and 10× Nupage reducing buffer (Bio-Rad). The protein gels were transferred onto polyvinylidine difluoride (PVDF) membranes for 90 min at 4 °C (110 V), blocked with 5% nonfat milk for 1 h at room temperature, and incubated overnight at 4 °C with primary antibodies against Cx43, Na_V_1.5 or α-tubulin in PBS-T with 5% nonfat milk. Acetylated α-tubulin and acetylated H3 were used as cytoplasmic and nuclear markers for acetylation. The membranes were washed 5 min × 4 with PBS-T, incubated with HRP labeled secondary antibody (1:5000) at room temperature in PBS-T with 5% nonfat milk for 30 min, washed again 5 min × 4 with PBS-T, and developed using the ECL™ Western Blot Detection Reagents (Bio-Rad). The image was detected using the Biorad imager. The density of the bands was quantified using ImageJ.

Primary antibodies used in this study include rabbit anti-Cx43 (AB1728, Merck Millipore, Billerica, MA, USA), mouse anti-Cx43 (AB1727, Merck Millipore), mouse anti-α-tubulin (# T5168, Sigma-Aldrich, St. Louis, MO, USA), rabbit anti-acetylated H3 (# 06-599, Merck Millipore), rabbit anti-acetylated-α-tubulin (BML-SA452-0100, Enzo Life Sciences, Farmingdale, NY, USA) and rabbit anti-Na_V_1.5 (# ACC-001, Alomone Labs, Jerusalem, Israel).

### 4.7. Statistics

Averaged values are presented as the Mean ± SEM. Statistical analyses were performed with the Normality and one-way ANOVA tests using the Bonferroni method in Origin 8.6. Data with *p* < 0.05 were considered significant.

## 5. Conclusions

This study demonstrates for the first time that class-selective HDAC inhibitors, entinostat and ricolinostat, produce no or moderate effects on functional electrical coupling and cardiac sodium currents in normal mammalian ventricular myocardium, which distinguished from pan-HDAC inhibitors. Therefore, class-selective HDAC inhibitors are likely to benefit broad patients with improved cardiac profile in clinical practice.

## Figures and Tables

**Table 1:** HDAC inhibitor Activity Assays.

HDACI	A_1_	C_1_ (nM)	IC_50_(1) (nM)	A_2_	C_2_ (nM)	IC_50_(2) (nM)	B	R^2^
TSA * (RFU)	2829 ± 219	10.6 ± 1.3	7.3 ± 0.9	298 ± 71	258 ± 76	179 ± 53	84 ± 7	0.98
VOR * (% RFU)	48.8 ± 7.7	140 ± 30	97 ± 21	44.1 ± 7.1	1086 ± 312	753 ± 216	6.1 ± 2.4	0.99
FK228 ^†^ (% RFU)	63.5 ± 5.3	7.9 ± 0.9	5.5 ± 0.6	27.7 ± 2.8	2524 ± 678	1749 ± 470	8.8 ± 1.4	0.99
LBH589 (RFU)	8322 ± 711	57.4 ± 22.2	39.8 ± 15.4	927 ± 357	2959 ± 838	2050 ± 581	374 ± 3	0.96
MS-275 (RFU)	20,111 ± 3080	629 ± 166	436 ± 115	20,106 ± 2150	12,926 ± 3655	8958 ± 2533	15,408 ± 1138	0.97
ACY-1215 (RFU)	14,914 ± 6840	16.9 ± 15.1	11.7 ± 10.5	32,744 ± 6835	204 ± 52	141 ± 36	3369 ± 263	0.99

* Adjusted values from data published in [13]; ^†^ adjusted values from data published in [14].

**Table 2:** Inhibitory constants for HDAC inhibitors used in this study.

HDACI	LBH589	LBH589	MS-275	MS-275	MS-275	ACY-1215	Tubacin	VPA
	(nM) [23]	(nM) [26]	(nM) [19]	(nM) [23]	(nM) [26]	(nM) [20]	(nM) [26]	(nM) [23]
HDAC	IC_50_	K_I_	IC_50_	IC_50_	K_I_	IC_50_	K_I_	IC_50_
HDAC1	3 ± 0	1.00 ± 0.1	180 ± 70	181 ± 62	22 ± 2	58	28 ± 4	1.584 × 10^+6^ ± 0.3
HDAC2	3 ± 0	0.65 ± 0.1	---	1155 ± 134	65 ± 5	48	42 ± 3.5	3.07 × 10^+6^ ± 0
HDAC3	4 ± 1	1.1 ± 0.15	740 ± 250	2311 ± 803	360 ± 15	51	275 ± 320	3.07× 10^+6^ ± 0
HDAC8	248 ± 11	105 ± 20	44,900 ± 18,100	>100,000	---	100	170 ± 10	7.442 × 10^+6^ ± 2.74
HDAC6	61 ± 1	1.50 ± 0.5	>100,000	>100,000	---	4.7	16 ± 2	>100,000
HDAC4	12 ± 5	550 ± 50	---	>100,000	---	7000	17,000 ± 2500	---
HDAC5	---	80 ± 10	---	---	---	5000	1500 ± 250	---
HDAC7	14 ± 7	4550 ± 315	---	>100,000	---	1400	8500 ± 1500	>100,000
HDAC9	3 ± 2	3200 ± 200	---	505 ± 37	---	>10,000	---	>100,000

>100,000 = incomplete inhibition at 100 μM [HDACI] [23]; VPA = valproic acid, another short chain fatty acid low-affinity HDAC inhibitor.

## Supplementary Materials

Supplementary materials can be found at http://www.mdpi.com/1422-0067/19/8/2288/s1.
