# Supplementary material for: Differences in Functional Expression of Connexin43 and NaV1.5 by Pan- and Class-Selective Histone Deacetylase Inhibition in Heart
Source: Int J Mol Sci. 2018 Aug 4;19(8):2288. doi: 10.3390/ijms19082288 (PMC6121244; doi:10.3390/ijms19082288)
Supplement: Supplementary file 1 [file ijms-19-02288-s001.pdf]

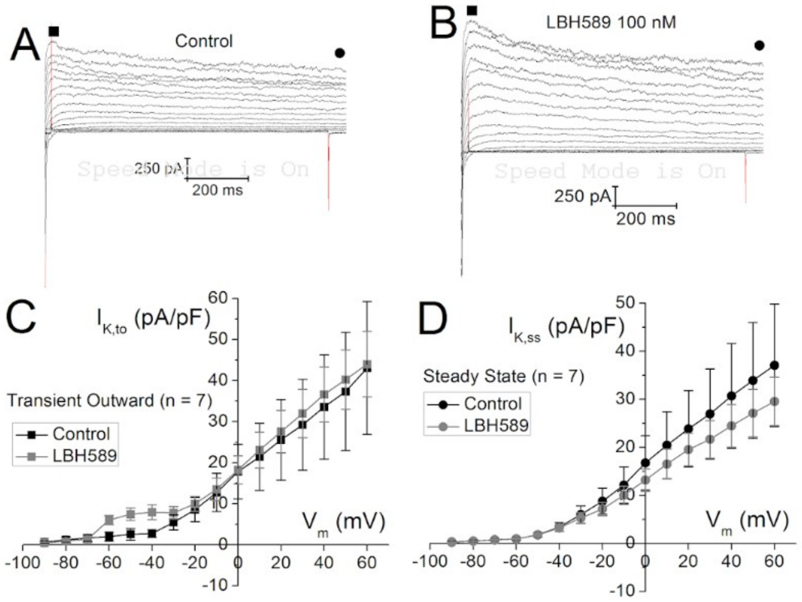

Figure S1. Transient and Steady State K^+ currents after 24 h treatment with 100 nM LBH589. (**A**) K^+ current traces in response to depolarizing voltage clamp steps applied to NMVMs under control conditions. (**B**) Similar K^+ current traces recorded from NMVMs after 24 h treatment with 100 nM panobinostat. (**C**) Peak transient outward K^+ current ($I_{K,to}$, ■)-voltage relationship obtained from NMVMs under control conditions or after 100 nM LBH589 treatment ($n = 7$, average ± SEM). (**D**) Steady state outward K^+ current ($I_{K,ss}$, ●)-voltage relationship obtained from NMVMs under control conditions or after 100 nM LBH589 treatment ($n = 7$, average ± SEM).

No significant changes in transient outward (■) and steady state (●) K^+ currents occurred after 24 h treatment with 100 nM LBH589 in NMVMs treated for 24 h with 100 nM panobinostat.

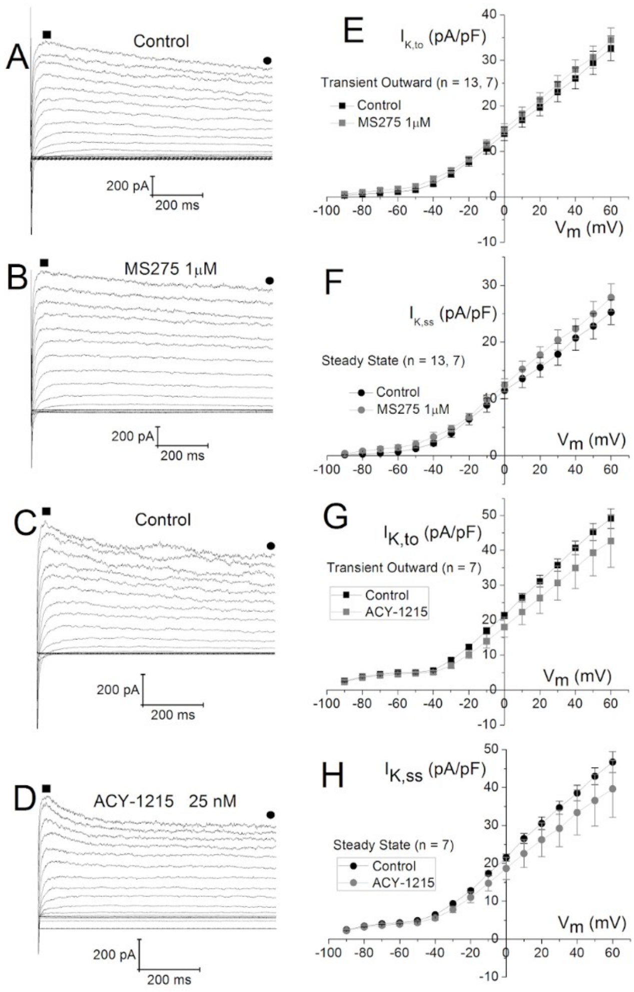

Figure S2. Transient and Steady State K^+ currents after class-selective HDAC inhibition. (**A**–**D**) K^+ current traces in response to depolarizing voltage clamp steps applied to NMVMs under control conditions and after 24 h treatment with 1 μM entinostat (MS275) or 25 nM ricolinostat (ACY-1215). (**E**–**F**) Similar K^+ current traces recorded from NMVMs after 24 hr treatment with 100 nM panobinostat. (**G**) Peak transient outward K^+ current ($I_{K,to}$, ■)-voltage relationship obtained from NMVMs under control conditions or after 100 nM LBH589 treatment (n = 7, average ± SEM). (**H**) Steady state outward K^+ current ($I_{K,ss}$, ●)-voltage relationship obtained from NMVMs under control conditions or after 100 nM LBH589 treatment (n = 7, average ± SEM).

Again, no significant changes in NMVM transient outward (■) and steady state (●) K^+ currents occurred after 24 h treatment with 1 μM MS275 or 25 nM ACY-1215 in NMVMs.
